# Screening for Cervical Cancer Using Automated Analysis of PAP-Smears

**DOI:** 10.1155/2014/842037

**Published:** 2014-03-20

**Authors:** Ewert Bengtsson, Patrik Malm

**Affiliations:** Division of Visual Information and Interaction, Department of Information Technology, Centre for Image Analysis, Uppsala University, Box 337, 751 05 Uppsala, Sweden

## Abstract

Cervical cancer is one of the most deadly and common forms of cancer among women if no action is taken to prevent it, yet it is preventable through a simple screening test, the so-called PAP-smear. This is the most effective cancer prevention measure developed so far. But the visual examination of the smears is time consuming and expensive and there have been numerous attempts at automating the analysis ever since the test was introduced more than 60 years ago. The first commercial systems for automated analysis of the cell samples appeared around the turn of the millennium but they have had limited impact on the screening costs. In this paper we examine the key issues that need to be addressed when an automated analysis system is developed and discuss how these challenges have been met over the years. The lessons learned may be useful in the efforts to create a cost-effective screening system that could make affordable screening for cervical cancer available for all women globally, thus preventing most of the quarter million annual unnecessary deaths still caused by this disease.

## 1. Cervical Cancer Screening

Cancer of the cervix uteri is the second most common cancer among women worldwide, with more than half a million new cases each year and about half as many deaths. The variation in incidence rate between countries is striking. In many countries it is the most common cancer among women while in some countries it is down at 10th place. About 86% of the cases occur in developing countries. In Africa the age-standardized incidence rate is 25 per 100,000 per year; in some countries on that continent it is more than double that rate. In India the rate is 27 while it is 5.7 in USA and 3.7 in Finland [1]. We thus see more than a factor of ten variations in cervical cancer incidence rates between the lowest and highest countries.

While a part of this variation may be attributed to general variations in living conditions and the spread of the Human Papillomavirus, HPV, in the population the major part is attributed to the success of screening using the Papanicolaou test (PAP-test). If detected early, cervical cancer is curable and the 5-year survival rate is as high as 92% [2]. The idea behind the PAP-test is that cellular changes that may develop into cancer are detected at such an early stage that they can be removed through a simple operation, thus preventing the cancer. Evidence for the importance of the PAP-test can be found in statistics from many countries where the PAP-test is used in systematic, comprehensive screening programs. In Sweden, for example, the overall incidence of cervical cancer declined by 67% over a 40-year period, from 20 cases per 100 000 in 1965 to 6.6 cases per 100 000 women in 2005. Detailed studies of the cancer statistics confirm this [3, 4].

### 1.1. The PAP-Smear

The original PAP-smear is produced in a very simple and straightforward way; a brush or spatula is used to gently scrape cellular material from the squamocolumnar junction in the cervix and this is smeared onto a glass slide of about 25 × 50 mm. The cells are stained, fixated, and then visually examined under a microscope. The test was first suggested by Papanicolaou in 1928 but it took almost 15 years before it was generally accepted by the medical community [5, 6]. A monograph in 1943 [7] gave a detailed account of how the screening should be conducted and this procedure has since been widely adopted, leading to the remarkable reduction in cervical cancer incidence mentioned in the previous paragraphs.

The screening is conducted by cytotechnologists, cytotechs for short, who through a light microscope examine the cell sample for signs of malignancy. Through this procedure they can not only find proof of invasive cancer but also detect certain cancer precursors, allowing for early and effective treatment. The cytotechs are laboratory technologists who go through a specialized training, typically of about one year. When they find something that looks suspicious for malignancy on a specimen it is reported. In many labs the finding is then confirmed by a cytopathologist, a medical doctor specializing in cellular pathology, who makes the final decision whether it is a (pre-)malignant lesion or not and thus takes the medical responsibility for the diagnosis. A detected high grade premalignant lesion typically leads to the woman being offered a colposcopy and, if a lesion is confirmed, an operation to remove it. The detection of a low grade lesion may lead to a follow-up smear being taken after a shorter time interval than the normal 2-3 years.

In principle, the screening task is straightforward. The morphological changes that a cell undergoes when it is being transformed into a malignant cell are quite apparent and easy to describe. The nucleus becomes larger and more irregularly shaped, the cytoplasm becomes smaller so that the nuclear/cytoplasm size ratio changes, and the chromatin distribution in the nucleus changes to become more coarse and irregularly distributed (see Figure 2).

To visually detect these changes we need to see details close to the optical resolution limit. A nucleus is around 10 microns in diameter and the chromatin structures and shape variations are at the micron or submicron level. Therefore a high power lens is used, typically 40x. The precancerous lesion may be quite small and local and the number of diagnostic (pre-)malignant cells on a specimen may be low. It is desirable to detect a precancerous lesion even if there are only a few diagnostic cells present on the specimen. This creates a demanding search problem. A smear covers about 25 × 50 mm and typically contains a few hundred thousand cells, sometimes even more. The screening is initially done at low resolution using a 10x lens, and when something suspicious is seen the screener switches to 40x. At 10x around 1,000 fields of view need to be scrutinized to cover the whole sample. The time required for this varies depending on how difficult the sample is, but on average it only takes 5–10 minutes. There are recommendations saying that, due to the hazards of fatigue, a cytotech should not work more than 7 hours a day and analyse no more than 70 samples [8]. Even when following this recommendation, the cytotech has to inspect three image fields per second on the average. Furthermore, since the visible precancerous changes may be quite local, the cytotech needs to maintain full concentration all the time in order not to risk missing some diagnostic cells.

## 2. Historical Development of Automated Screening Systems

Based on the fact that the changes in cell morphology are quite obvious and the fact that the visual screening is very demanding, tedious, and expensive in terms of labour requirements, there were very early, only a decade after the PAP-test became generally accepted, proposals for automating the screening through some kind of scanning and image analysis mechanism [9]. The hope was that an automated system would be able to do the screening both at a lower cost and with higher accuracy.

Since then a large number of projects have attempted to develop screening systems. The problem turned out to be a lot harder than anticipated. It took more than 40 years before the first successful commercial systems appeared. And still automated screening is not sufficiently cost-effective to completely replace the visual screening judging from the relatively limited penetration of automated screening systems in the screening operations worldwide. In this section, we briefly outline this development and try to see for each new generation of systems in what ways they improved on earlier systems, what were the main problems, and what was learned. We also discuss the underlying technical aspects and try to understand what makes the problem so hard and how one can go about solving it.

### 2.1. First Generation Systems

The Cytoanalyzer project in the US was the first attempt at building an automated screening device for PAP-smears [10]. The system was based on the concept that cancer cells could be distinguished from normal cells on the basis of nuclear size and optical density. The system included automatic slide feed and autofocus circuits. The image analysis was based on hard-wired analogue video processing circuits that generated two-dimensional histograms of nuclear size versus nuclear optical density. The spatial resolution was 5 micrometers. Preliminary experiments had shown that it was possible to detect the difference in size between normal and malignant cells at this resolution. This was the first fully automated microscope and as such a quite expensive project. Unfortunately, tests with the Cytoanalyzer revealed that the special purpose fixed logic pattern recognition produced too many false alarms on the cell level [11]. There were numerous objects of a size similar to malignant cells present also on normal specimens, for example, clumps of blood cells, strands of tissue and mucus, overlapping epithelial cells, and so forth. Every sample, including the normal ones, was thus found to be suspicious for abnormality. The project failed in the early sixties, mainly because of this artefact rejection problem.

Due to the bad reputation for cytology automation caused by this early and expensive failure in the US, the attempts at automation over the next couple of decades were shifted to Europe and Japan. In Britain a one-parameter (nuclear size) automatic screener was developed in the late sixties [12]. It failed for the same reason as the Cytoanalyzer.

In Japan, Watanabe and coworkers at Toshiba developed CYBEST [13]. Their first version used special-purpose electronic circuits while later versions were based on general purpose digital computers, thus bridging the gap between old analogue and new digital technology. The pixel size was around one micron. They extracted four different features from the cell images: nuclear area, nuclear density, cytoplasmic area, and nuclear/cytoplasmic ratio. They also realized that nuclear shape and chromatin pattern were useful parameters but were not able to reliably measure these features automatically mainly because the automatic focusing was unable to reliably produce images with all the cell nuclei in sufficiently good focus. The chromatin pattern measure that was proposed by this group was the number of blobs within the nuclear region. Four generations of prototype systems were developed over a 15-year period. The last one used strobed illumination and nonstop scanning motion to reach high scanning speeds. The prototypes were used in large field trials in the Japanese screening program and showed promising results but none of them became a product [14].

### 2.2. New Generations of Systems

When the first generation systems were developed there were no interactive computers and no display units capable of showing digital images available. This, of course, made development much harder. However, during the seventies it became possible to develop interactive image analysis systems, albeit with very limited capacity, typically with a memory size of a few hundred kB and a monochrome or binary display. These systems were used to explore new image segmentation, feature extraction, and classification designs which led to a new generation of systems in the early 1980-ies such as BioPEPR [15], FAZYTAN [16], Cerviscan [17], LEYTAS [18], and at the authors' laboratory the Diascanner [19].

Typical cellular features used in these systems were similar to those used by CYBEST, although there were many variations in exactly how the features were extracted. The most important factor was found to be that the cells were digitized at sufficiently high resolution and in better focus. In order to be able to scan a whole specimen sufficiently rapidly while still being able to do the crucial analysis at high resolution, some of these designs, for example, the Diascanner, used a dual resolution approach, an initial low resolution search scan followed by high resolution scans of fields of interest. Most of these systems reached an operational prototype stage in the mideighties. Some of the systems reported classification accuracies that were well within the range of what is achieved by the conventional visual screening. But none reached the market, and an important reason for this was lack of cost effectiveness; automated microscopes and computers with sufficient processing power were still too expensive.

The progress in computer display technology, that had been important in making it possible to create interactive systems that could be used for developing new automated screenings systems, eventually also led to the possibility of developing interactive screening systems. For the early systems the only option was full automation, or possibly stopping the automated microscope to physically show an operator the cell that was suspected as being abnormal. The concept was to create a “prescreening” system; that is, a system that for a reasonably large fraction of specimens would be able to say that they are perfectly normal and could be classified as such without any human inspection. All other specimens, on which the system found something that indicated that they might not be normal, would have to be screened in the conventional fully manual way. In the late eighties, computer displays and memories had reached sufficient capacity to make it feasible to save images of suspicious cells that were good enough for a human to judge whether the object could be a malignant cell or something else. The PAPNET system from Neuromedical Systems was the first to introduce interaction into automated screening [20]. After an initial low resolution object search, high resolution fields were processed, first by an algorithmic classifier and then by a neural network classifier. The output of the classifiers was a ranking of the abnormality of the detected “cells,” so that images of the 64 most abnormal ones could be stored on a magnetic tape and later shown to the cytotech at a review station. There the decision whether the specimen should be classified as normal or suspicious was taken. For the suspicious cases a cytopathologist would do the final analysis and make the decision whether the woman should be called for follow-up or not.

In the late eighties there was a great increase in interest in cytology automation in the US for economic/legal reasons and many new projects were started [21]. One new aspect that appeared at this time was new ways of preparing the samples. The Cytyc Corporation had developed their own automated specimen preparation technique, ThinPrep, which based on liquid cytology made much cleaner specimens than the conventional smears, at the expense of significantly more complex preparation technique [22]. Another similar preparation method was developed by AutoCyte [23].

The AutoPap 300 from NeoPath was similar to PAPNET in that it used conventional Pap-smears and neural network classifiers [24]. It increased the image acquisition rate by utilizing strobed illumination similar to the CYBEST system. This was used at two resolution levels, an initial low resolution mapping of the specimen, followed by a high resolution field by field analysis of the most “interesting looking” parts of the specimen in a way similar to the earlier generation Diascanner. The image processing was carried out in custom designed processing boards. Most of the processing was based on mathematical morphology operations resulting in as many as 68 different features being sent to the classifiers. The final result was a “normal” versus “requires visual inspection” decision on the specimen level; that is, no interactive confirmation was used of the machine decision for the negative cases.

### 2.3. The First Commercially Available Systems

During the nineties there was strong competition between the American companies developing screening technology as well as struggles to get the various solutions approved by the powerful Food and Drug Administration, FDA. Screening systems were classified in a category of medical devices requiring premarket approval, meaning that no system can be sold in the US without FDA approval. Hundreds of millions were spent on developments and field trials and there was a shake-out; the companies merged and were acquired by larger companies. The first company with a screening product to finally receive FDA approval was Tripath in 1998. It was the merger of NeoPath, Neuromedical, and AutoCyte. The Tripath Company was in turn acquired by BD in 2006 and the system renamed BDFocalPoint Slide Profiler [25]. It is to a large extent based on the AutoPap 300 system. A new liquid based specimen preparation technique called SurePath has been added to further improve the system performance although it can also analyse conventional smears. According to the FDA approval, the system can be used to recognize about 25% of the slides as normal for no further review; the other 75% are ranked into five categories at risk for abnormality. There is also a possibility of visually reviewing fields of particular interest at a special review station. The system can also be used for quality control and claims increased sensitivity in detecting abnormalities [26].

Cytyc was quite successful with their improved liquid based preparation technique and could demonstrate better performance for that technique as compared to conventional smears. They also developed an interactive system with a computer prescreen that selected the most abnormal looking objects on each specimen for human inspection. In 2003 they received FDA approval for their ThinPrep Imaging System [27], and in 2007 they became part of the Hologic Company. The system is marketed for increasing detection of abnormalities by improved specimen preparation and screening both visually and by machine [28].

## 3. The Technical Challenges

In the quick review of the historical development above we have briefly mentioned some of the key features of the different generations of systems. We will now return to the different crucial aspects of the technologies behind a screening system and discuss what needs to be achieved in order to screen a sample in a time comparable to that of a human screener that is less than 10 minutes.

### 3.1. Specimen Preparation

**Figure 1:**
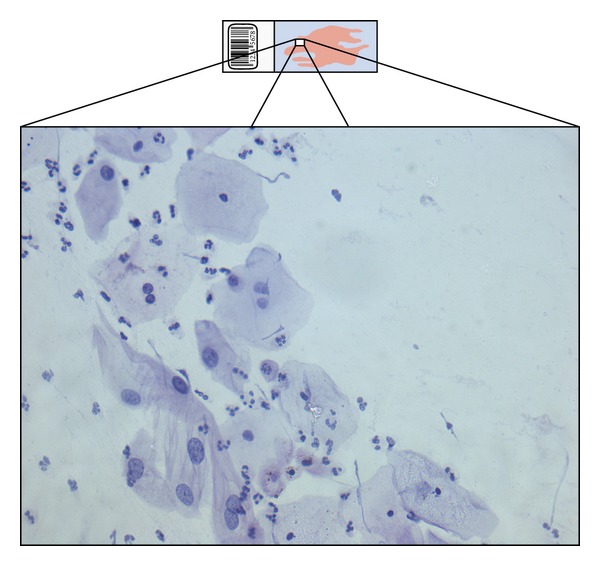
A typical PAP-smear and a high resolution field of view through a 40x lens. Approximately 10.000 such fields of view are needed to cover the whole slide.

**Figure 2:**
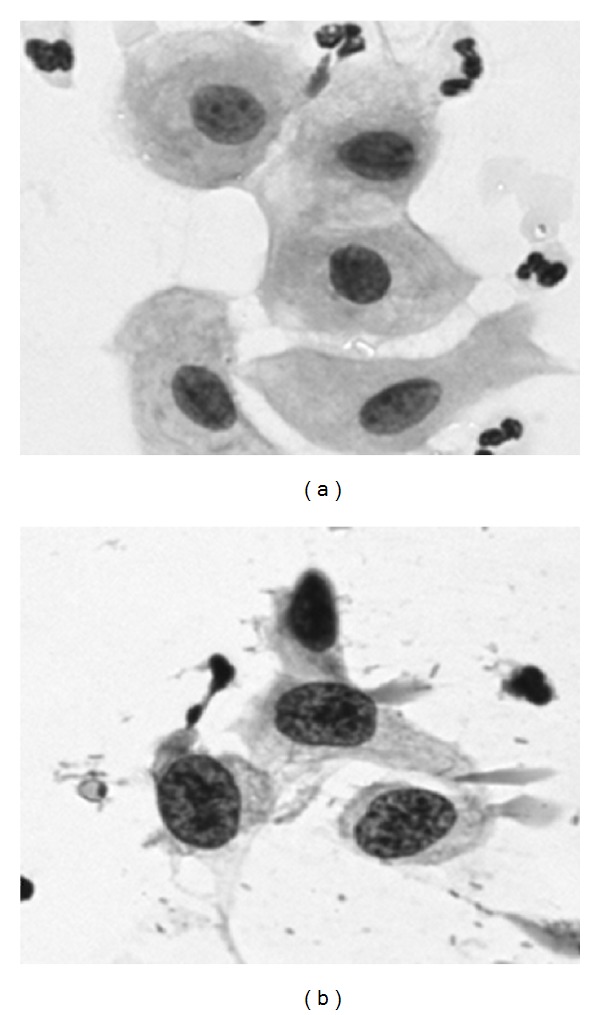
To the left a few normal cells and to the right some clearly atypical, premalignant cells.

**Figure 3:**
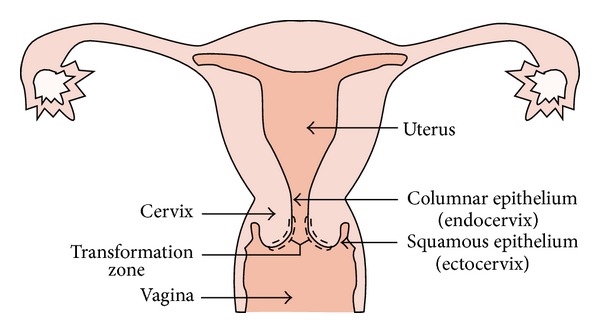
Illustration showing the anatomy of a uterus. For specimen acquisition it is important that cells are acquired from both the endocervical and ectocervical regions, that is, both above and below the region known as the transformation zone.

In the original PAP-smear the cellular material is manually spread over the glass slide. It is important that both the endocervical and ectocervical regions (see Figure 3) are represented in the sample and through the smearing there may actually be a mapping between the source region and the location on the slide. [29]. The samples are fixed and stained in a rather straightforward procedure, which can be done fully manually or in staining machines with varying degrees of sophistication. The material cost for the whole preparation is quite low, on the order of 1-2 US dollars. In Figure 1, an image of a high resolution field from a PAP-smear is shown.

The manual smearing and staining do unfortunately lead to big variations in specimen quality. Sometimes the cellular material may be unevenly distributed leading to dense clumps which light cannot penetrate while other parts of the slide may be empty. Even when the smear is done well there will still be regions which are too dense and have too many overlapping cells for reliable interpretation. An experienced cytotech can cope with great variations in specimen quality and still make a rather reliable assessment of the specimen, but the smears are very challenging to analyse automatically.

To make specimens that are better, both for visual and machine analysis, various liquid based cytology (LBC) preparation techniques have been developed. The common strategy here is to submerge the brush or spatula with all the cellular materials collected from the cervix in a liquid, which then is treated in various ways before it is deposited onto a glass slide, fixed, and stained. The result is ideally a cellular sample that is spread in a monolayer with optimal density over a well-defined part of the glass slide. The goal of this procedure is that the resulting samples should be easier to interpret reliably visually and in particular by machines. Several different techniques for liquid based preparations have been developed over the years, the two leading techniques are Surepath [25] and Thinprep [27] mentioned above. There have been numerous studies comparing the liquid based preparations to the conventional smears and most of them come to the conclusion that they are at least as good or better when it comes to reliability of detecting abnormalities [28, 30–32]. All currently marketed machine screening systems work with liquid based preparations.

The great disadvantage of the liquid based preparations is the associated operational costs. They require significantly more materials to be used for preparing a specimen, for example, vials, liquids, filters, and also more complex equipment, for example, centrifuges. The procedures are proprietary and the necessary equipment is sold as kits which increases the cost of preparing a slide to at least 10 US dollars. This causes significant economic problems in regions with limited resources. Still there are studies indicating that liquid based preparations are more effective [33, 34] while a large metastudy concluded that they could see no significant differences [35]. There are also alternative liquid based preparations that have been developed and are competing with lower costs [36, 37] although those have so far not been tested as extensively as the leading techniques.

### 3.2. Scanning

In order to analyse a cell sample in a computer, it should be scanned at sufficiently high resolution to reliably extract the features that can determine whether it is normal or indicating a precancerous change. This is very challenging. At a pixel size of 0.2 microns, a smear of 25 × 50 mm will give 31 billion pixels. Just transferring this amount of data from the camera to the computer will take minutes, even with the latest high-speed transfer techniques. Since there are no lenses that can resolve the whole specimen area at once and no image sensors with 31 gigapixels, we have the problem of repositioning the lens over a large number of image fields that together cover the specimen. A high resolution microscope lens gives a field of view with a diameter of around 0,5 mm and with a matching 6 megapixel sensor we will get 5000 image fields. Repositioning and capturing an image at each of these will take at least 10 minutes. This can be reduced by using nonstop motion and flash illumination to freeze the images. The CYBEST4 system was the first screening system to use this idea [14] and later it was used in the AutoPap [24]. An alternative is to use a 1D sensor with a length of, for example, 2000 pixels and smoothly move the microscope stage in the orthogonal direction. The Cerviscan [17] and Diascanner [19] systems used this idea. It is also used in the currently popular slide scanners by Aperio [38] although at a lower resolution.

**Figure 4:**
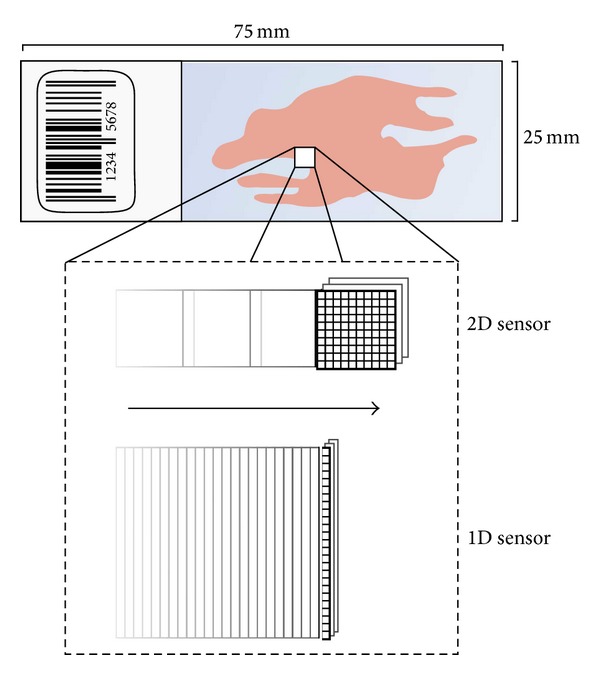
Illustration schematically showing image scanning using one- or two-dimensional sensor arrays. With a 2D array an image or a stack of images at different focus levels are read before the microscope stage moves a few hundred micrometers to a new position where this process is repeated as soon as the vibrations caused by the move have died out. With a 1D array the microscope stage is moving continuously and single lines in the direction orthogonal to the move are read into the computer creating a continuous flow of image data.

Another serious issue is focusing. In order to reliably extract the texture information from the cell image they must be in very good focus which requires high quality autofocus, which also is time consuming. An alternative is to scan the specimen at several focus levels and choose the best for each cell, which reduces the need for autofocus but increases the amount of data even more. So in summary it is quite demanding to scan a whole smear in a sufficiently short time at sufficiently high image quality. In Figure 4, an illustration of the two different scanning approaches mentioned above is seen.

One way to decrease the demands is to use a smaller part of the slide surface for the specimen. With a smear this cannot be done without decreasing specimen sampling quality. With liquid based preparation the area of the sample is around 1/10 of that of a smear, a great advantage when it comes to scanning. For smears we can instead use a dual resolution approach mimicking the way cytotechs switch between 10x and 40x lenses. If we scan with 1 micron pixel size we can cover the smear in 200 fields which can be done in about 20 seconds. This will produce a map of where cellular material with a suitable density is distributed, which can then be used to control where a number of scans at high resolution are acquired. A variant of this approach is to not only look for areas with suitable density of cells but also for areas with cells that look suspicious for abnormality. This dual resolution screening approach was first proposed by Poulsen [39] and used in some of the early systems, for example, the Diascanner [19]. A potential risk with this design approach is that, if the low resolution scan systematically misses some type of abnormalities, those abnormalities never become subject to the high resolution analysis.

### 3.3. Segmenting Cells and Nuclei

In order to extract the features describing the cells we must find and delineate each cell and/or cell nucleus in the specimen image. This is called image segmentation and is a crucial step in almost all image analysis based systems. Segmenting nuclei in PAP-smears is made very difficult by the same complications that make the smears hard for humans to analyse, that is, variable smear thickness and staining intensity, obscuring elements, and so forth. The earliest systems used thresholding based on greyscale for the segmentation in the very first systems using a fixed threshold value but later on with a value determined by histogram analysis as originally suggested by Prewitt and Mendelsohn [40]. More recent projects have used more complicated approaches. Bergmeir et al. [41] use mean shift and morphological filtering and later try Canny edge detection followed by the randomized Hough transform [42]. Bamford and Lovell [43] use a dual active contour algorithm. Malm and Brun [44] use Canny edge detection followed by anisotropic curve closing. In a recent review [45], five different classes of approaches to cell segmentation are identified and it is demonstrated how they have appeared and gained popularity over the years. There is still a need of developing new methods, since none of the existing ones are as flexible and robust as the human visual system in really identifying where the nuclear or cytoplasmic border is located in difficult cases.

The main requirement for a good cell nucleus segmentation method is that it accurately can detect and delineate the cell nucleus under different staining conditions and in the presence of disturbing object in the direct vicinity. A second important requirement is that this segmentation can be done quickly. We cannot spend more than a few milliseconds per cell if we are to accomplish the analysis in an acceptable time. The increasing computer power has made it possible to do this even with somewhat complex algorithms. It does, however, require the algorithms to be implemented in an efficient way, for instance, taking advantage of the possibilities of parallelism possible in modern computers.

### 3.4. Artefact Rejection

The goal for the segmentation algorithms is to find and accurately delineate cell nuclei (and sometimes cytoplasms) that are sufficiently well preserved and imaged to allow accurate extraction of features for the subsequent classification. But it will fail sometimes either because the image of the nucleus is corrupted by overlaying objects or other artefacts or when the cell is so poorly preserved or presented in the image that the extracted outline of the object will be wrong. It is then very important that we can detect this failure and discard the data from the object. Otherwise it will lead to unreliable classification performance on the specimen level. The process of analysing the segmentation results in order to remove erroneous results is called artefact rejection.

**Figure 5:**
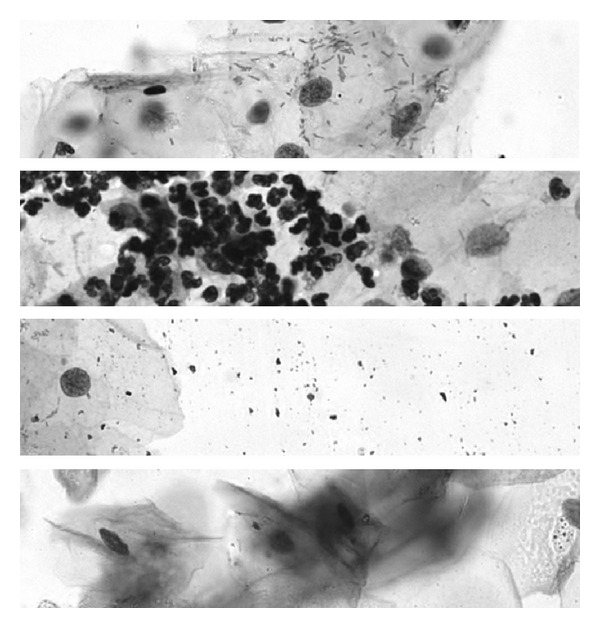
Images displaying four common types of artefacts found in PAP-smears. From top to bottom: bacteria, leucocytes, stain residues, and overlapping and folded material.

Artefact rejection is a difficult topic because there are an infinite variety of ways in which blood cells, inflammatory cells, folded and distorted cells, overlapping objects, mucus, staining mistakes, and so forth, influence the image of a cell (see Figure 5). But it is an absolutely essential step in a screening system. The motivation for this can be found in the statistics we have to deal with. A standard PAP-smear may typically contain 100,000–200,000 cells of the relevant cell types and we should be able to call it positive if we find 10–20 diagnostic, premalignant, or malignant cells (ideally a single clearly malignant cell should be enough). A classifier that only makes one percent false positive error will call at least 1000 cells positive even on a healthy sample, making every sample called positive and the system thus useless. One approach to deal with this problem is to make the classifier highly asymmetrical between false positive and false negative, that is, allowing it to miss-classify a large fraction of the actually malignant cells as normal. This may seem to defeat the purpose of the system which is to detect (pre-)malignancy. But the highly unbalanced numbers work that way. If the system has a false negative rate of 80% it will still detect 2–4 of the diagnostic malignant cells if we have 10–20 available. This is acceptable as long as the false positive rate is virtually zero, less than 0.001%. Creating such a classifier is hard but possible if we can work with perfectly imaged cells with accurate segmentation and carefully extracted features. To make sure this is the case we need very effective artefact rejection.

There is very little explicit research done on artefact rejection for cervical screening. Some research papers ignore the problem by working on visually selected or verified images of nuclei thus relying on manual artefact rejection, which of course cannot be done for a real screening system. Other papers include the artefact rejection in the segmentation or classification steps. Still, analysing the artefact rejection problem on its own makes it easier to see what performance can be achieved and to relate that to what is needed. Malm et al. recently presented such a study where they demonstrated a specificity of 99.38% on smears and 99.83% on LBC specimens, while maintaining a sensitivity of around 98% based on a material of around 12,000 automatically detected and segmented images of objects visually classified into cell nuclei and artefacts [46]. With that kind of performance we would still have a few hundred artefacts corrupting the data if we analyse 100,000 objects, so it may be hard to achieve the sensitivity of detecting a few abnormal cells without getting too many false positive samples. Still it points in the direction of what is necessary to achieve for a useful system.

### 3.5. Feature Extraction

When we have an accurate segmentation of cell nuclei, we can extract features describing the size, shape, and texture of the object. The most obvious features are those representing the greater size and more irregular overall shape of the malignant nuclei. Those features can be extracted at relatively low resolution and even without having the cell in perfect focus. Assuming perfect artefact rejection those features may be useful in detecting a large proportion of the clearly malignant cells and specimens. They were used in the first generation systems, which failed because of the lack of adequate artefact rejection. Over the years, many more features have been invented and tested. In [47] the different kinds of features that have been proposed were reviewed and systematically categorized.

The most important information about whether the nucleus is normal or (pre-)malignant is found in the chromatin pattern or texture of the nucleus. The DNA in the nucleus is distributed in a different way when the cell is influenced by a malignant process. This effect can be seen and measured even with PAP-stain which is not stoichiometric for DNA. Measuring the chromatin distribution is, however, difficult. The most common approaches are based on a statistical description of neighbouring grey levels typically measured through so-called transition probability matrices as originally proposed in [48]. Another approach is to see the individual chromatin granules as objects that are segmented, and then the spatial relations between these objects are described, for example, through graph analysis methods [49]. Different ways of measuring chromatin features are discussed in [50] and also in [47]. A very important aspect of the chromatin analysis is that you need perfect focus and very high quality images to reliably represent this pattern which is at or beyond the optical resolution limit.

## 4. Classification Strategies

The ultimate goal of the PAP-smear screening process is to find women with precancerous lesions, so that they can be treated before the malignancy develops into potentially lethal invasive cancer. When running the conventional visual screening process, the cytotechs can classify most specimens as clearly normal and needing no further review. It is important to realize that we typically are screening a general population so that the great majority of samples, perhaps 96%, are normal. Still some specimens look more suspicious and are referred to a cytopathologist for review; in some cases the malignancy may be so obvious that the cytotech can be sure about it; still the confirmation by a pathologist is required. A positive sample will then lead to the women being called in for additional investigation, possibly involving colposcopy and a biopsy, and if the lesion is confirmed, a simple operation with a loop electrosurgical excision procedure or similar to remove it. There are different levels of changes in cell appearance that can be detected and there is a consensus standard for how to classify these called the Bethesda system [51]. According to the Bethesda system there is also a range of abnormalities from the perfectly normal slide via slight abnormalities ASC-US, low grade lesions, LSIL, high grade lesions, HSIL, and finally cancer. It is of course particularly important to pick up the higher grades and not clear whether it really is necessary to detect the slight changes. Not all low grade lesions will progress to cancer even when left untreated and when they do it may take a decade or even more. With a regular recurring screening program taking a new sample with a few years interval, the probability of detecting a lesion before it progresses to cancer is therefore high even if the risk of missing it at a single screening occasion is rather high, perhaps 20–30%.

When adding an automated screening device to the overall screening setup, it can be used in various ways. The original concept was to do an automated prescreening, which would be able to say that a substantial fraction of the specimens were normal while having very few false negatives, that is, not missing any, or very few true positive specimens. To reach a low false negative rate a relatively high false positive rate could be accepted since those specimens were screened visually. Even if only 50% of the specimens could be dismissed as clearly normal the system would remove half the visual screening workload and could be cost-effective if it did not add too much to the overall screening cost. The SurePath system is typically used in this mode and set to only remove 25% of the specimens, while also ranking the positives into different categories of likelihood of being truly positive.

Another way of using an automated system is to run it in parallel to visual screening. Since humans and machine most likely will make different errors, the combined system will increase the sensitivity of the overall screening process, that is, reduce the false negative rate. But the downside is that the overall workload and cost are increased rather than decreased. The Thinprep system is mainly marketed to be used in this mode.

### 4.1. The Rare Event Approach

An image analysis based automated screening system analyzes cells one by one and can, based on the features extracted from the cell image, classify it as being normal or abnormal. The simplest way of using the information from the cell classifier is to simply count how many abnormal cells we have found on the specimen and if it is over a low threshold we call the specimen suspicious. The problem is to set the threshold so that we do not miss true positives in particular not high grade ones, while avoiding too many false positives [52].

This approach disregards the information about how certain the cell classifier is about its decision. It may be that one cell is found to be clearly malignant while another one is very close to the threshold for being normal. If we retain this information, we can make a specimen level decision about whether we have found too much abnormality to call the specimen normal, either based on just a few clearly malignant cells, or a larger number of cells slightly over the threshold [53].

If we can do the image analysis and feature extraction online as we scan the specimen, we may stop the analysis as soon as we have found sufficient evidence that the specimen is not clearly normal. This may save time by making it unnecessary to scan and process the rest of the specimen. This strategy is not controversial since it does not increase the risk of false negatives. But since the great majority of specimens are normal in a typical screening situation we would need to be able to stop early also when we have found sufficient evidence that a specimen is normal in order to really save time. And this is controversial; it is generally required that a cytotech looks at the entire specimen before calling it normal. Still only a small part of the cells scraped from the cervix really makes it onto the glass so we are not analysing all possible cells even when we look at the whole slide. However, with a conventional PAP-smear there is a kind of mapping between areas in the cervix onto the slide so we may systematically miss some important region by stopping early. For a liquid based preparation, much fewer cells are available for analysis on the specimen, but there is a mixing step involved so we can assume that we have a random sample and can stop as soon as we have sufficiently many cells for a required statistical significance in the decision function.

### 4.2. Malignancy Associated Changes (MAC)

In the approach to the screening problem described so far the systems have been mimicking the way humans do it, that is, searching for potentially rare (pre-)malignant cells. Achieving a low false negative rate even for specimens with a low number of diagnostic cells is challenging and requires analysing very many cells. There is, however, an alternative approach based on so-called malignancy associated changes (MAC). It was discovered already in 1967 that cells in the vicinity of a malignancy are influenced so that they undergo small, often subvisual changes in the chromatin texture [54]. These discoveries were confirmed in the early research on automated cervical screening [55, 56]. Even though these shifts were not strong enough to be useful on the individual cell level, it made it possible to detect abnormal specimens through a statistical analysis of the feature distributions of a small population, a few hundred cells, provided that these features were extracted very accurately. Since these changes are present in all cells in a large neighbourhood of a malignant process we can have a different approach to the screening. We need to be able to reliably detect these subtle changes in cell populations from a specimen. But we will not need to search through the whole specimen; only data from sufficiently many cells to characterize the chromatin distribution of the cell population is needed, typically around 500 cells. The group that has been pursuing this idea most systematically is the one at the British Colombia Cancer Research Centre [57, 58]. Also in this case we need very accurate artefact removal; we do not want to extract texture data from artefacts. And we need perfect focus for each cell. Even a small deviation from perfect focus causes significant changes in the chromatin image. It has not yet been convincingly demonstrated that MAC alone can detect early premalignant changes with sufficient sensitivity.

### 4.3. The DNA Ploidy Approach

The malignant process not only modifies the distribution of DNA in the nucleus, it also increases the amount of DNA. With a stoichiometric stain, a histogram over the integrated optical density of all the nuclei will show a diploid distribution for normal cells and a different aneuploid distribution for malignant cells. Therefore modified, stoichiometric PAP-like stains have been developed and used for automated screening studies [59–61], showing quite promising results. This method is currently being used in China in a study involving several hundred thousand women [62]. Since this approach is based on densitometric measurements, there are rather strong requirements of consistent staining and control of the illumination and calibration of the imaging. The artefact rejection is also very important; we must be sure that the DNA measurements are only from single, free-lying, well-preserved nuclei. A significant problem with these modified stains is to get them accepted by the wider community, since new appearance of the samples may require expensive retraining. This concept can also be used with the conventional PAP-stain, but due to the lack of stoichiometric staining the ploidy measurements will be less reliable.

### 4.4. Field Test Statistics and Performance Requirements: Technical and Ethical Issues

The statistics needed for developing a successful screening system is difficult on several levels as described in the previous paragraphs. But we also have difficult statistics on the highest population screening level. A screening machine that systematically misses a significant proportion of the positive samples will decrease the confidence in the screening programs and put the women at risk of developing invasive cancer before the problem is detected. Since a screening system is typically used mainly to analyse normal specimens, at most a few percent of the samples are truly positive. To prove the detection capabilities of the system with high confidence we need it to analyse hundreds of positive cases. In a development phase we can achieve high numbers of positive cases by selectively running positive cases in the machine. But for the final evaluation we should run it on the typical mix of routine specimens. We thus need a volume of tens of thousands of specimens to properly test the system. And the situation is made even more complicated by the fact that there are various kinds of rare abnormalities that are detected by the visual screening. We need to verify that the machine does an acceptable job also for those. During this testing phase the machine will have to be used in parallel to visual screening, causing double operational costs plus extra work for doing the comparisons and statistical evaluations. The final system verification stages are thus a difficult and expensive threshold to get over before a new system is ready for widespread use.

What performance requirements a screening machine must meet is an issue that has caused controversy over the years. The perfect machine should have zero percent false negatives and zero percent false positives. In practice this is impossible so we have to consider what the realistic requirements are.

The false negative rate is primarily an ethical issue. If we miss to detect high grade lesions, it may lead to the women getting cancer. The requirement should then be that the machine is at least as good as the current visual screening process. But there are two aspects also of this requirement. It should on average not miss more specimens than what is missed by a good cytotech. Additionally it should not systematically miss any relevant kind of lesion.

The false positive rate of the initial automated specimen inspection on the other hand is an economic issue. False positives from the whole screening setup, after inspection by a cytologist are expensive and cause anxiety for the woman who is called for a new investigation which may be interpreted as a message that she may have a cancer. But no screening system is set up so that the machine positive samples lead to a call for the woman to come to a new examination. The samples classified as potentially positive by the machine are screened visually by a cytotech and, if still found to be positive, by a cytologist. This rescreening costs money and reduces the gain of having the machine screening. Still very high levels of “machine false positives” can be accepted, for example, 75% for one of the commercial systems. The machine is then set up so that only clearly normal specimens are classified as normal, and everything else should be inspected also by a human.

### 4.5. Performance Requirements: Legal Issues

The deployment of an automated screening process is made significantly more complicated by the legal aspects. There are in most countries, for good reasons, strong regulations for how to test a screening machine before it is approved for routine use. In the USA a screening machine needs premarket approval by FDA before it can be sold for clinical screening use. Obtaining such approval involves detailed documentation of all aspects of the machine as well as extensive testing of its performance in large, well-documented studies. But the legal aspect is not limited to obtaining approval from the appropriate authorities. If a machine misses to detect a high grade lesion present in a sample and this leads to a woman getting cancer, the manufacturer of that machine can be sued for the damages caused. This can be extremely expensive and is a risk no manufacturer can take. Therefore the procedures for how to use the screening machines are designed to minimize this risk. One way of doing this is to require a human screener to visually inspect some data from every specimen, for example, a set of images of objects the machine determined to be the most “malignant looking” on the specimen or a number of selected image fields optically through the eye-piece of the microscope. Thus the responsibility of calling the specimen “normal” is transferred from the manufacturer to the user. Another approach has been to run the machine screening in parallel to conventional visual screening with the rationale that the errors done by machine and by the human are different and thus the overall sensitivity for detecting malignancy is increased. By setting the threshold for when to call a specimen “normal” without further human inspection very conservatively some manufacturers have decreased the risk of missing a positive specimen to the point where it has been deemed acceptable. In the USA that threshold currently seems to be at the 25% level; that is, 75% of all specimens will have to be screened both by machine and humans. No fixed such thresholds are set in other parts of the world.

All these legal precautions are meant to protect the women from unnecessary risks of obtaining cancer in spite of having been screened, which of course is a good thing. But they have also significantly contributed to the fact that automated screening so far has failed to have a real impact on the screening costs. Still the majority of women in the world are not offered regular screening because of the high associated costs. An automated screening system that is almost as good as the best visual screening systems at a significantly lower cost could save millions of women from dying in cervical cancer. But will such a device ever be accepted by the legal systems? It will not probably be accepted in the USA but perhaps in some other parts of the world.

## 5. Conclusion

We have in this paper outlined the 60-year history of efforts to automate the screening for cervical cancer and pointed out how the different generations of systems have tried to meet the challenges of this difficult task. We have also discussed the different aspects of these challenges and how they can be met. Now in conclusion let us discuss where we stand today.

The purpose of an automated inspection system is to decrease the cost and/or false negative rate of a screening program. To achieve the first goal it is necessary that the cost of operating the system including capital and maintenance costs in addition to the direct operational costs is less than what it costs to do the same work as the system does with conventional manual methods. It is doubtful if the present generation commercial systems meet these goals. They have been more focused on the second goal. By running machine screening in parallel to visual screening it is likely that the machine misses other abnormalities than the human screener, thus reducing the overall false negative rate. The overall operational cost will however be higher. The currently available commercial systems may thus marginally increase the quality of the screening but they will not significantly decrease the cost.

It is today known that cervical cancer is caused by human papillomavirus infection. An alternative or supplementary screening method is to test for such infections. There are studies showing that combining both kinds of analysis adds sensitivity of detecting precancerous lesions [63]. Still there seems to be limited advantage of replacing the PAP-test by only a virus test. The knowledge that the cancer is caused by a virus infection has also opened up the possibility of vaccination against the HPV virus. If such vaccination programs became globally comprehensive, the prevalence of the cancer could be decreased to the level where screening would be no longer necessary. But unfortunately it is not likely that that will happen any time soon and it will take decades even after everyone is offered vaccination before the effects reach all age groups.

The historical developments of the screening field have taken place in parallel to the fantastic development of computer technology. We now have millions of times more computer power available per dollar than we had when the first digital screening systems were built. Similarly the image sensor technology has developed dramatically. Even since the time the first version of the current commercial systems was developed some 15 years ago there has been significant progress in the underlying technologies. There have also been significant developments on the algorithmic side, although perhaps not as dramatic as for the hardware. All this sets the stage for a good opportunity today to develop a really cost-effective screening system. It is most likely that a fully automated screening system today can be built at a cost of at least an order of magnitude lower than the cost of the currently available commercial systems. Such a system could make it economically feasible to implement comprehensive screening systems also in the poorer parts of the world and eventually have an impact on the high incidence of cervical cancer there. There are of course major challenges in organizing an effective screening program in such countries, but a compact, robust automated screening system could make a big difference.
